# Effect of the Leaving Group and Solvent Combination on the LFER Reaction Constants

**DOI:** 10.3390/ijms13022012

**Published:** 2012-02-13

**Authors:** Mirela Matić, Sandra Jurić, Bernard Denegri, Olga Kronja

**Affiliations:** Faculty of Pharmacy and Biochemistry, University of Zagreb, 10000 Zagreb, Ante Kovačića 1, Croatia; E-Mails: mmatic@pharma.hr (M.M.); sjuric@pharma.hr (S.J.)

**Keywords:** reaction constant, nucleofuge specific parameter, nucleofugality, electrofugality, solvation, solvolysis

## Abstract

Fine effects that influence the variations of the reaction constants *s*_f_ in LFER log *k* = *s*_f_(*N*_f_ + *E*_f_) have been summarized here. Increasing solvent polarity in the series of binary mixtures increases the solvolysis rates for the same factor for all benzhydryl derivatives in which the solvation of the leaving group moiety in the transition state is substantial, *i.e.*, log *k vs. E*_f_ correlation lines are parallel (same *s*_f_). For the substrates in which the demand for solvation of the leaving groups moiety is reduced, (e.g., carbonates) *s*_f_ parameters decrease as the fraction of the water in a given solvent/water mixture increases (log *k vs. E*_f_ plots converge), due to decreasing solvation of the electrofuge moiety toward bigger electrofugality. The abscissa of the intersection of the converging plots might indicate the critical electrofugality above which the solvolysis rates should not depend of the water fraction. Larger reaction constant *s*_f_ indicate later transition state for structurally related substrates only, while *s*_f_ parameters for structurally different substrates cannot be compared likely due to different intrinsic barriers. Inversion in relative abilities of leaving groups is possible if they have similar reactivities and are characterized with different reaction constants.

## 1. Introduction

Numerous investigation of the leaving group abilities (nucleofugality) in S_N_1 reactions have been reported in chemical literature, but the comprehensive nucleofugality scale have been developed only recently [1,2]. Two major problems had been responsible for the lack of general nucleofugality scale. First, relative nucleofugalities depend on the substrate structure as well as on the nature of solvent. Thus, different demand for solvation of various leaving groups (LG) in the ground and transition state [3–23], electronic and steric effects in the ground state, are all rate determining factors. The second major problem was that the reaction rates could reliably be obtained by conventional methods in relatively narrow range of reactivities (between 10^3^ and 10^−5^; 8 orders of magnitude). Therefore, even though the first problem could have been overcame by defining the nucleofugality scale that refers to a given substrate (electrofuge) and a given solvent, the narrow accessible reaction range allowed only a limited number of leaving groups to be examined. This range can only moderately be extended by variation of temperature, as was done by Noyce, who used 1-phenylethyl as an electrofuge [24].

Analogously to the previously established model for construction of the most comprehensive nucleophilicity/electrophilicity scales [25,26], in collaboration with Mayr’s group we applied for the first time a linear free-energy relationship (LFER) approach based on benzhydryl derivatives for developing the nucleofugality scale [1,2]. Application of the series of benzhydryl derivatives instead of a single structure enabled measuring the solvolysis rates by conventional methods for wide spectrum of nucleofuges by combining poor leaving groups (weak nucleofuges) with highly stabilized benzhydrylium ions (good electrofuges) in a substrate, or good leaving groups (good nucleofuges) with destabilized benzhydrylium ions (poor electrofuges).

**Scheme 1:**
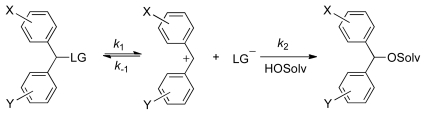
Solvolysis of benzhydryl derivatives.

Accordingly, nucleofugality/electrofugality scales have been developed, based on studying the solvolysis rates of a large variety of X,Y-substituted benzhydryl substrates with different leaving groups in various solvents (Scheme 1). The absolute rate of the S_N_1 solvolytic reaction can be estimated with high precision for benzhydryl derivatives, and with reasonable accuracy for other types of substrates, using the following three-parameter LFER equation [1,2]:

(1) $$\text{log }k=s_{\text{f}}(E_{\text{f}}+N_{\text{f}})$$

in which: *k* is the first-order solvolysis rate constant (s^−1^), *s*_f_ is the nucleofuge-specific slope parameter, *N*_f_ is the nucleofugality parameter, *E*_f_ is the electrofugality parameter. Such an approach separates the contributions of an electrofuge and a nucleofuge in overall solvolytic reactivity. The nucleofugality parameter (*N*_f_) corresponds to leaving group ability in a given solvent, while the electrofugality parameter *E*_f_ is an independent variable that refers to the ability of the carbocation generated in the heterolysis reaction (S_N_1) to leave the nucleofuge. A set of substrates comprising 39 benzhydrylium ions as reference electrofuges and 14 leaving groups in the various series of solvents (in total 101 reference nucleofuge/solvent combinations) has been selected to obtain the reference *E*_f_ and also *N*_f_ and *s*_f_ parameters by optimization procedure according to Equation (1). Predefined parameters for optimization were: *E*_f_ = 0.00 for dianisylcarbenium ion (X = Y = 4-OCH_3_) and *s*_f_ = 1.00 for chloride nucleofuge in pure ethanol. In this special type of the LFER, nucleofugality (*N*_f_) of a given leaving group is defined as the negative intercept on the abscissa of log *k* (25 °C) *vs. E*_f_ plot, which is in most cases close to the experimentally accessible reaction rate, so the long range extrapolation as a source of large error is omitted.

Nucleofugalities of numerous additional leaving groups have been determined from the log *k vs. E*_f_ linear correlations, using as substrates benzhydryl derivatives with the reference electrofuges [27–31]. The electrofugality scale has also been extended to other electrofuges that are not benzhydrylium derivatives [32,33], whose *E*_f_ parameter were calculated from the first-order solvolysis rates and the *N*_f_ parameters of the leaving group used. According to existing electrofugality and nucleofugality scales the solvolysis rates can be predicted in the range of 25 orders of magnitude. The practical detailed guide for estimating the solvolysis rates is published recently [1].

The concepts of electrofugality and nucleofugality have also been examined theoretically [34]. Reasonably good correlation has recently been found between the electrofugality paramameters (*E*_f_) and the theoretical electrofugality indexes (*ν*^−^) for the referent benzhydrylium ions [35]. In addition, intrinsic nucleofugalities for numerous leaving groups have been calculated by using theoretical background that has not included heterolytic transition states [36].

It should also be mentioned that Bentley proposed the electrofugality parameters to be defined from the solvolysis rates of chlorides in 80% aqueous ethanol, and proposed a modified equation for calculating the reaction rate, assuming *s*_f_ = 1 [37].

**Figure 1:**
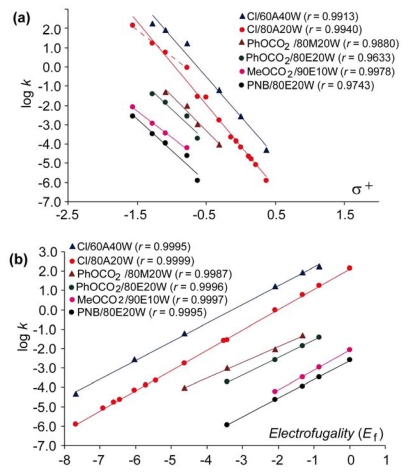
Comparison of the Hammett-Brown correlation (**a**) and the correlation according to Equation 1 (**b**) of the series of electrofuges with various nucleofuges in different solvents.

If one compares the Hammett-Brown correlation (2) with rearranged Equation 1 (1a):

(1a) $$\text{log }k=s_{\text{f}}E_{\text{f}}+s_{\text{f}}N_{\text{f}}$$

(2) $$\text{log }k=\rho^{+}\sigma^{+}+\text{log }k_{0}$$

it is evident that the fundamentals of *E*_f_ parameters are the same as the fundamentals for *σ*^+^ values. Consequently, the reaction constants (the slope parameters *σ*^+^ and *s*_f_) measure the same phenomenon. Indeed, *E*_f_ parameters correlate reasonably well with *σ*^+^ values (*E*_f_ = −4.39∑*σ*^+^ −6.14, *r* = 0.996), hence it turned out that the relation between *σ*^+^ and *s*_f_ is roughly *ρ*^+^ = −4.4*s*_f_ [2]. However, the Hammett-Brown correlation is poor if it is applied to the unsymmetrically substituted benzhydryl derivatives (and other α-*R*-diarylmethyl derivatives) due to non-additivity and non-linearity [38]. Even though Hammett-Brown correlation is much more widely applicable as far as structural variation is concerned, while Equation (1) is limited so far to benzhydryl derivatives only, the advantages of the latter is that all correlations between log *k* and *E*_f_ are, regardless of the leaving group structure, quite good in all cases used without exception (mostly *r* > 0.999). Because of that, fine effects that change the trends and the values of the *s*_f_ parameters can be observed, which could not be observed earlier using the Hammett or Hammett-Brown correlation. Figure 1 compares the Hammett-Brown correlation (a) and the above LFER approach based on Equation 1 (b) by plotting the rates for solvolysis of some benzhydryl derivatives with different leaving groups in various solvents against the *σ*^+^ and *E*_f_, respectively. The plots and the correlation coefficients presented on Figure 1 show that the correlations are much better if Equation (1) is applied, so the use of the *s*_f_ values here instead of the *ρ*^+^ for evaluation of the results obtained with benzhydryl substrates is justified. Plots for chlorides on Figure 1a indicate that the magnitude of *ρ*^+^ may depend on the region of electrofugalities of substrates. Thus, the electrofuges without alkoxy substituents produce steeper plots than those with alkoxy substituents (red dashed line). On the other hand, the slopes obtained with the same substrates using the above mentioned LFER approach do not depend on the structure of substrates, but all points unambiguously belong to the same correlation line, indicating that advantageously only a narrow range of electrofuges is necessary to obtain reliable correlations and the corresponding reaction constants *s*_f_ (Figure 1b).

In this mini-review we summarize some major phenomena observed from the values and variations of the reaction constants *s*_f_ which are as follow: (a) indication of an earlier or later transition state; (b) dependence of the reaction constant on the fraction of water in a series of organic solvent; (c) inversion of reactivities of leaving groups; and (d) possible design of the substrate whose solvolytic reactivity does not depend on the fraction of water in a given solvent. The reaction constants *s*_f_ (calculated from rate constants obtained at 25 °C) for numerous leaving groups in a given solvents are presented in Table 1.

## 2. Indication of Earlier and Later Transition State

The Hammett-Brown *ρ*^+^ parameter has often been rationalized as a measure of the positive charge in the transition state. Accordingly, the magnitude of *s*_f_ parameter should directly indicate earlier or later TS of a given substrate, which is not always the case. Mayr showed that the backward combination reaction (k^−1^ on Scheme 1) for chlorides (in 80% aqueous ethanol) proceeds without the barrier for substrates that produce less stabilized benzhydrylium ions than that in which X = Y = Me, *E*_f_ = −3.5 (Scheme 1), *i.e.*, the processes are diffusion controlled [39]. On the other hand, most of the LGs presented in Table 1 are weaker nucleophiles that react for several orders of magnitude slower than chloride [31,40], hence the backward combination reactions proceed via barriers if the elelctrofugality is *E*_f_ > −3.5. The transition states for those heterolysis reactions are inevitably less carbocation-like than those for chlorides despite their lower reactivity. Deviations from Bell-Evans-Polanyi principle and the Hammond postulate can be rationalized in terms of lower intrinsic barrier for benzhydryl chlorides than for other less reactive leaving groups. However, while the *s*_f_ parameters for chloride are about *s*_f_ ≈ 1 in most solvent used (Table 1), many reaction constants obtained for reaction that proceed via earlier TS (e.g., data for DNB, PNB, *etc.*) are *s*_f_ > 1, clearly showing that smaller or larger *s*_f_ values do not simply indicate earlier or later transition states, *i.e.*, terms early and late transition states are misleading, when related to Hammond’s postulate. We are not yet able to rationalize the factors responsible for observation that *s*_f_ > 1, but important influence might come from differences in intrinsic barriers of leaving groups.

Reaction constants *s*_f_ for structurally very close substrates, whose solvolytic behavior is in accord with Bell-Evans-Polanyi principle and Hammond postulate, can indeed be considered as the indication of the more or less carbocation-like TS. For example, systematically higher reaction constants in all solvents for less reactive methyl carbonates than those for phenyl carbonates show that methyl carbonates solvolyze via more carbocation-like TS in which the charge separation is more advanced. The same has been observed in preliminary investigations of fluorinated benzoates. Thus, the more reactive pentafluorobenzoates produce plots with lower *s*_f_ (0.87 in 70% aqueous ethanol and 0.90 in 80% aqueous ethanol) than 2,4,6-trifluorobenzoates (0.94 in 70% aqueous ethanol and 0.98 in 80% aqueous ethanol), which are less reactive for about one order of magnitude [41]. On the other hand, chlorides and bromides solvolyze without the backward barrier in the range in which kinetic data have been collected, and in accordance with above consideration, produce correlation plots with essentially same slope (Table 1).

## 3. Variation of the Rate Constant with Solvent Polarity

The increase of the fraction of water in a given organic solvent (ethanol, methanol or acetone) does not influence the reaction constant *s*_f_ for numerous benzhydryl derivatives, so they either produce parallel log *k vs. E*_f_ lines or the slope parameters differ in the limits of experimental error. Almost parallel correlation lines have been observed for substrates that have halogens as a leaving groups [32] (Exceptions are the plots obtained with chlorides in aqueous acetonitrile and acetone in which the decrease of *s*_f_ with solvent polarity have been observed), aliphatic fluorinated esters (trifluoroacetate and heptafluorobutyrate) [27], and acetate [1]. In other words, the increasing solvent polarity increases the solvolysis rates for the same factor of all benzhydryl-LG derivatives, regardless of the structures. Having in mind that the correlation lines are obtained with the substrates that have the same leaving group, while the structures of the electrofuges differ, it appears that the dominant electrostatic solvation effects are directed toward leaving group. It is not surprising, since in benzhydryl moiety the positive charge is considerably delocalized, and the demand for solvation is small in comparison to the demand for solvation of the leaving group moiety. The reaction constants of numerous leaving groups in various solvents are presented in Table 1.

Charged substrates, such are sulfonium salts (dimethyl sulfonium and tetrahydrothiophenium salts have experimentally been examined) that generate neutral leaving groups, also produce parallel lines (Table 1) [29,30]. Their solvolytic behavior can similarly be rationalized as above. The major factor that controls the variation of the rates with solvent composition is the solvation of the positively charged substrate in the ground state. Small differences of solvation due to different electrofuges are negligible in comparison to the solvation of the positive charge located mostly on the sulfur atom. Also, small shifts of the transition state toward the substrate in faster reactions or toward the benzhydrylium ion in slower reactions have little effect on the overall stabilization by solvation, so the increase in water content decreases the reactivity of all substrates for the same factor, *i.e.*, practically the same *s*_f_ parameters are obtained in all solvents used.

**Figure 2:**
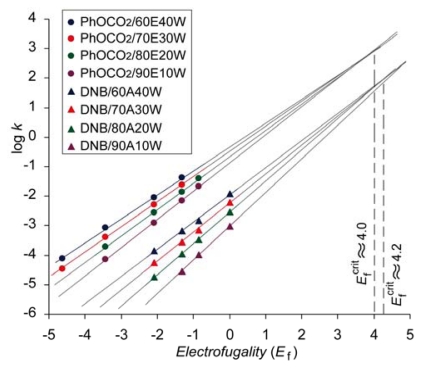
Plots of log *k vs. E*_f_ for the solvolysis reactions of X,Y-substituted benzhydryl phenyl carbonates and 3,5-dinitrobenzoates in aqueous ethanol and aqueous acetone, respectively.

Carbonates (phenyl and methyl carbonate) were the first cases where the convergence of the log *k vs. E*_f_ lines has been observed in the series of aqueous organic solvents [42]. The log *k vs. E*_f_ correlation lines for phenyl carbonates obtained in ethanol-water binary solvents are presented on Figure 2. In our further investigations the same phenomena have been observed for 3,5-dinitrobenzoate (DNB) [43] (Figure 2) and 2,4-dinitrophenolate (DNP) [28]. Preliminary results obtained with fluorinated benzoates and fluorinated phenolates also show that the same phenomenon occurs [41]. It should be mentioned that the trends presented in Figure 2 do not depend on the validity of Equation (1) and that the same patterns of converging plots would be obtained if instead of the electrofugality parameter, e.g., the log *k* of the corresponding chlorides in ethanol have been used.

Convergence of the log *k vs. E*_f_ plots have been rationalized in terms of less demand for solvation of the leaving group moiety in the transition state. If the solvation of the nucleofuge moiety in the TS is diminished for any reason, differences in solvation of various electrofuge moieties might have observable influence on the overall reaction rate. Increasing electrofugality in the series of given benzhydryl derivatives (carbonates or benzoates) comes from more extensive positive charge delocalization and thus demand for solvation of the TS is reduced by more polar solvent. On the other hand, in the case of benzhydryl substrates with weaker electrofuges, the demand for solvation is bigger since the positive charge is less dispersed from the cationic center. Thus, polarity of solvent has a bigger impact on the reactivity for the substrates that produce less stabilized benzhydrylium ions than for those that produce more stabilized benzhydrylium ions. This consideration is in accord with experimental findings [42,43]. For example, the ratio between the solvolysis rate constants of benzhydryl phenyl carbonate in which X = Y = CH_3_ in Scheme 1 (*E*_f_ = −3.44) in 60% aqueous ethanol and 90% aqueous ethanol is 11.8, while that of the substrate in which X = MeO and Y = CH_3_ (*E*_f_ = −1.32) in the same solvents is only 5.7. The net result of decreasing reaction rate ratios in more polar and less polar solvent with increasing electrofugality is convergence of the log *k vs. E*_f_ plots.

The structural features responsible for diminished solvation of carbonates are different from those responsible for DNB. The negative charge generated in heterolysis of phenyl and methyl carbonates is distributed almost equally over all three oxygen atoms because of the resonance and the negative (inverse) hyperconjugation effects. Quantum-chemical calculations indicated that the NBO charges at B3LYP/6-311++G(d,p) level in phenyl carbonates anion are −0.70, −0.73 for oxygen atoms involved in resonance, and −0.66 for that attached to phenyl and involved in inverse hyperconjugation (−0.80, −0.77 and −0.66 for methyl carbonate ion), supporting the intense negative charge delocalization and therefore diminished demand for solvation of the leaving groups moiety in the TS [42]. In DNB, in addition to delocalization of the negative charge by resonance of the carboxylate moiety, further dispersion of the charge in the TS occurs caused by polar effects of the nitro-substituents, which can account for diminished solvation. Also, relatively large hydrophobic aromatic surface causes small demand for solvation.

Position of the transition state of solvolysis might also cause convergence of the log *k vs. E*_f_ plots, due to less demand for solvation in the earlier transition state of more reactive substrates [23]. Since substrates that produce less stabilized benzhydrylium ions in solvolysis proceed via later, more carbocation-like TS, the solvent polarity should enhance the reactions more than those that proceed via earlier TS. The convergence of the plots would, therefore, be in a same fashion than that described above caused by diminished solvation of the leaving group moiety in the TS. This behavior would be expected for substrates for which the backward reaction (*k*^−1^ on Scheme 1) proceeds through barrier. The fact that parallel lines have been observed in solvolysis of acetates, which is so far the least reactive leaving group and whose combination barrier is rather high in the experimental range [1,40], indicate that the influences of the position of the TS on convergence of the plots are rather small. Parallel slopes observed for fluorinated carboxylates support the assumption that the shift of the TS toward carbocation-like structures for substrates that produce more stabilized electrofuges does not cause substantial decrease of *s*_f_ if the fraction of the water increases in a given organic solvent [27].

## 4. Inversion of Relative Reactivities of Leaving Groups

**Figure 3:**
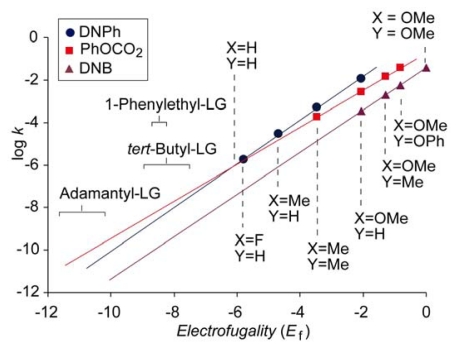
Comparison of log *k vs. E*_f_ plots for 2,4-dinitrophenolate and phenyl and methyl carbonate in 80% aqueous ethanol at 25 °C.

The relative reactivity of two leaving groups can be decided by comparing the nucleofugality parameters. However, if the nucleofugalities of two LGs are close enough in magnitude, while the *s*_f_ parameters differ substantially, a simple comparison of nucleofugalities may be misleading because of the possible intersection of the log *k vs. E*_f_ plots in the range of electrofugalities that corresponds to real stable structures that solvolyze in the range of experimental reactivities. Figure 3 shows the log *k vs. E*_f_ plots for phenyl carbonate (PhOCO_2_, *N*_f_ = −0.74, *s*_f_ = 0.90), 3,5-dinitrobenzoate (DNB, *N*_f_ = −1.43, *s*_f_ = 0.98) and 2,4-dinitrophenolate (DNP, *N*_f_ = 0.22, *s*_f_ = 1.03) obtained in 80% aqueous ethanol. The plots that correspond to DNB and DNP do not intersect in the experimental range, indicating that all possible DNPs, regardless of the electrofuges, solvolyze faster than the corresponding 3,5-dinitrobenzoates. However, the abscissa of the intersection of the plots for DNPs and phenyl carbonates is in the region of stable structures. Accordingly, for more reactive substrates, DNPs solvolyze faster than phenyl carbonates, but for less reactive substrates, *i.e.*, those with weaker electrofuges, such as adamantyl, *tert-*butyl or 1-phenylethyl [32], carbonates may be slightly more reactive than the corresponding DNPs. It should be emphasized that the value of the abscissa of the intersection of the plots for DNP and PhOCO_2_ is reliable because it is not obtained after far extrapolation but fells in the range of experimental data, and also because the correlations of both plots are very good (*r* > 0.999).

## 5. Critical Electrofugality

Converging log *k vs. E*_f_ plots intersect in the region of higher electrofugalities (*E*_f_ between 3 and 6) [43]. Figure 2 shows the extrapolated plots that correspond to solvolysis of DNB in aqueous acetone and phenyl carbonates in aqueous ethanol. One can speculate that the region of critical electrofugality *E*_f_^crit^ might exist, above which the solvolysis rates in a given binary solvent series do not depend of the water content, *i.e.*, of the solvent polarity. It is, for example, indicated on Figure 2 that for phenyl carbonates *E*_f_^crit^ ≈ 4, hence the substrate producing carbocation with that electrofugality would solvolyze in the series of aqueous ethanol via same barrier (*ca.* 14 kcal mol^−1^). Similarly, log *k vs. E*_f_ plots for DNBs in the series of aqueous acetone intersect in a similar region of electrofugality (Figure 2), indicating that the barrier for DNB that produce carbocation with *E*_f_ ≈ 4.2 is the same (*ca.* 14.7 kcal mol^−1^), regardless of the water fraction.

The Grunwald-Winstein equation which correlates the reaction rates of a given substrate in various solvents and the above LFER equation which correlates the rates of series of substrates in a given solvent are different but complementary approaches. Because of that, additional support for the decreasing sensitivity of the carbonates and DNBs toward solvent polarity with increasing electrofugality has been demonstrated by Grunwald-Winstein *m* parameters obtained from various solvent-ionizing power scales. For example, while in aqueous ethanol *m*_OTs_ = 0.64 for benzhydryl phenylcarbonate in which X = Y = Me, for substrate in which X = Y = MeO it is reduced to *m*_OTs_ = 0.33 [43]. Critical electrufugalities, obtained from *m*_OTs_ *vs. E*_f_ plots by extrapolating the plot to *m*_OTs_ = 0.00, have practically the same values as those obtained above from the intersection of the log *k vs. E*_f_ plots (4.0 ± 0.0 for PhOCO_2_ in aqueous ethanol and 4.2 ± 0.0 for DNB in aqueous acetone).

Taking that the concept of critical electrofugality is feasible, it can, for example, be predicted that the solvolysis rates of benzhydryl DNB in which X = N(Me)_2_ and Y = H (*E*_f_ = 2.38) (Scheme 1) still increase with increasing fraction of water in aqueous acetone, while the rates for DNB in which X = Y = N(Me)_2_ (*E*_f_ = 4.84) are the same regardless of the solvent polarity, *i.e.*, they do not depend on the solvent content. However, the assumption that substrates with highly stabilized electrofuges solvolyze with the same rate regardless of the fraction of the water is still to be proved experimentally.

## Figures and Tables

**Table 1:** Reaction constants *s*_f_ (nucleofuge-specific parameters) for some leaving groups in various solvents.

Solvent a	Leaving Group b,c						

	Cl	Br	PhOCO_2_	MeOCO_2_	HFB	TFA	DNB
TFE	0.85	0.95					
100M	0.99	0.99	0.90	1.01	0.90		
90M10W	0.99	0.99	0.85	0.97	0.89	0.84	
80M20W	1.00	1.00	0.81	0.94	0.84	0.83	
70M30W					0.84	0.81	
60M40W					0.84		
100E	1.00	0.93			0.93	0.89	1.09
90E10W	0.98	0.93	0.96	0.98	0.88		1.06
80E20W	0.99	0.95	0.90	0.95	0.88	0.82	0.98
70E30W	0.96	0.96	0.85	0.93	0.86	0.84	
60E40W	0.97		0.81	0.89	0.86	0.82	
90AN10W	1.08						
80AN20W	1.00						
60AN40W	0.96	0.99					0.97
90A10W	1.11	1.01				0.97	1.13
80A20W	1.05	0.90			0.91	0.88	1.10
70A30W	1.00	0.95	0.88	0.94	0.91	0.88	0.98
60A40W	0.97	0.97	0.83	0.88	0.88	0.86	0.90
50A50W	1.03	0.93	0.77	0.86	0.87	0.81	

**Solvent**	**Leaving Group**^b^,^c^						

	**PNB**	**AcO**	**Me_2_S**	**THT**^d^	**DNP**^e^	**OTs**	**OMs**

TFE						0.94	1.00
100M			0.89	0.86	1.03	0.82	
90M10W					0.97		
80M20W			0.89	0.86	0.94		
70M30W							
60M40W			0.85				
100E			0.87	0.86	1.06	0.78	0.80
90E10W					1.02		
80E20W	0.95		0.86	0.85	1.03	0.80	0.84
70E30W					0.99		
60E40W			0.86				
90AN10W							
80AN20W	0.98	1.11					
60AN40W	0.91	1.08				0.82	0.83
90A10W	1.17				1.16	0.89	
80A20W	1.16	1.18			1.10	0.83	0.84
70A30W					1.02		
60A40W	1.11	1.17			0.98		
50A50W					0.91		

a Binary solvents are v/v at 25 °C. A = acetone, E = ethanol, M = methanol, TFE = 2,2,2-trifluoroethanol, AN = acetonitrile, W = water;

b PhOCO_2_ = Phenyl carbonates, MeOCO_2_ = Methyl carbonates, HFB = Heptafluorobutyrates, TFA = Trifluoroacetates, DNB = 3,5-Dinitrobenzoates, PNB = 4-Nitrobenzoates, AcO = Acetates, Me_2_S = Dimethyl sulfide, THT = Tetrahydrothiophene, DNP = 2,4-Dinitrophenolates, OTs = *p*-Tosylates, OMs = Mesylates;

c s*_f_* parameters are taken from ref. [1] unless otherwise specified;

d s*_f_* parameters are taken from ref. [30];

e s*_f_* parameters are taken from ref. [28].
